# Multiphysics model of a rat ventricular myocyte: A voltage-clamp study

**DOI:** 10.1186/1742-4682-9-48

**Published:** 2012-11-21

**Authors:** Abhilash Krishna, Miguel Valderrábano, Philip T Palade, John W

**Affiliations:** 1 Department of Electrical and Computer Engineering, Rice University, 6100 Main Street, Houston, 77005, USA; 2 Methodist Hospital Research Institute, Methodist DeBakey Heart & Vascular Center, 6565 Fannin Street, Houston, 77030, USA; 3 Department of Pharmacology and Toxicology, University of Arkansas for Medical Sciences, 4301 West Markham Street, Little Rock, 72205, USA

## Abstract

**Background:**

The objective of this study is to develop a comprehensive model of the electromechanical behavior of the rat ventricular myocyte to investigate the various factors influencing its contractile response.

**Methods:**

Here, we couple a model of *Ca*^2 + ^dynamics described in our previous work, with a well-known model of contractile mechanics developed by Rice, Wang, Bers and de Tombe to develop a composite multiphysics model of excitation-contraction coupling. This comprehensive cell model is studied under voltage clamp (VC) conditions, since it allows to focus our study on the elaborate *Ca*^2 + ^signaling system that controls the contractile mechanism.

**Results:**

We examine the role of various factors influencing cellular contractile response. In particular, direct factors such as the amount of activator *Ca*^2 + ^available to trigger contraction and the type of mechanical load applied (resulting in isosarcometric, isometric or unloaded contraction) are investigated. We also study the impact of temperature (22 to 38°C) on myofilament contractile response. The critical role of myofilament *Ca*^2 + ^sensitivity in modulating developed force is likewise studied, as is the indirect coupling of intracellular contractile mechanism with the plasma membrane via the *Na*^ + ^/*Ca*^2 + ^exchanger (NCX). Finally, we demonstrate a key linear relationship between the rate of contraction and relaxation, which is shown here to be intrinsically coupled over the full range of physiological perturbations.

**Conclusions:**

Extensive testing of the composite model elucidates the importance of various direct and indirect modulatory influences on cellular twitch response with wide agreement with measured data on all accounts. Thus, the model provides mechanistic insights into whole-cell responses to a wide variety of testing approaches used in studies of cardiac myofilament contractility that have appeared in the literature over the past several decades.

## Background

Cardiac muscle contraction is a result of a transient increase in myoplasmic *Ca*^2 + ^ concentration *Ca*^2 + ^_*myo*_. Sarcolemmal (SL) membrane depolarization triggers *Ca*^2 + ^influx via dihydropyridine (DHP)-sensitive L-type *Ca*^2 + ^channels. Following diffusion across a small sub-membrane dyadic space, this influx activates ryanodine receptors (RyRs) controlling ryanodine-sensitive *Ca*^2 + ^release channels in the junctional portion of the sarcoplasmic reticulum (jSR). Fabiato and Fabiato [1] named the process calcium-induced calcium release (CICR). *Ca*^2 + ^subsequently diffuses from the dyadic space into the myoplasm. Ultimately, myoplasmic *Ca*^2 + ^ concentration *Ca*^2 + ^_*myo*_ is returned to resting levels by combination of: (a) *Ca*^2 + ^buffering in the dyadic space and myoplasm; (b) sequestration of *Ca*^2 + ^y by sarcoplasmic/endoplasmic reticulum *Ca*^2 + ^-ATPase (SERCA)-type calcium pumps lining the longitudinal portion of the sarcoplasmic reticulum (LSR); and (c) *Ca*^2 + ^ extrusion from the myoplasm by *Na*^ + ^/*Ca*^2 + ^exchangers and *Ca*^2 + ^-ATPase pumps on the sarcolemmal membrane.

*Ca*^2 + ^ is an extremely important and highly versatile second messenger in cardiac cells, which plays a crucial role not only in excitation-contraction (E-C) coupling but also in excitation-transcription coupling [2]. Various inter-connected *Ca*^2 + ^signalling pathways help preserve the integrity of the cellular *Ca*^2 + ^ system despite any disturbances (e.g., changes in stimulation frequency or inotropic state). A key role for the dyadic *Ca*^2 + ^release system is the formation of an adequate myoplasmic *Ca*^2 + ^ transient that can serve as an input driving signal for the intracellular contractile machinery (the myofilaments). The resultant contractile response is conditioned by a number of additional factors that include the mechanical load; sarcomere equilibrium length; myofilament *Ca*^2 + ^sensitivity; and the temperature. Although it is well-known that the contractile response of a cell is a function of the stimulation frequency (its force-frequency response (FFR)), this study is limited to an investigation at 5 Hz (unless otherwise specified), a physiologically relevant heart rate for a rat ventricular myocyte.

## Computational methods

**Figure 1:**
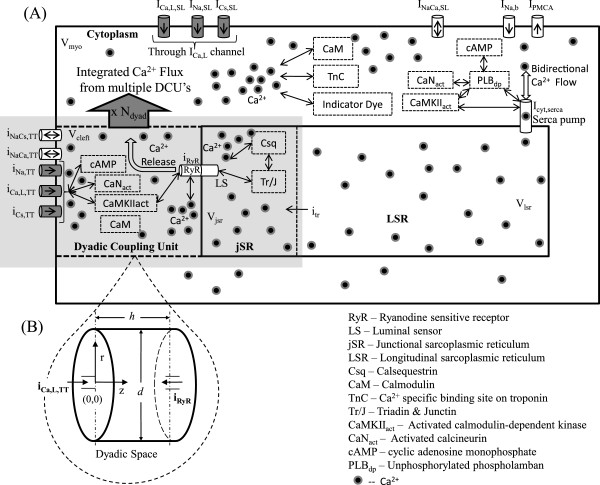
**Cellular fluid compartments.** (**A**) Model configuration showing dyadic space, jSR, LSR, myoplasm and SL; (**B**) Inset provides a more detailed description of the dyadic space showing the coupling of the two types of *Ca*^2 + ^channels (trigger and *Ca*^2 + ^release channels) via the dyadic fluid medium. Only one representative dyadic coupling unit is shown; however the whole model contains 10,000 such identical units lumped together. The region showing interaction of the DHP-sensitive *Ca*^2 + ^channel (I_*Ca,L*_) with the junctional sarcoplasmic reticulum (jSR) via the dyadic coupling unit (DCU) is highlighted (grey).

All simulations and analysis were performed on a 2.8GHz Intel^*®*^ Core^TM^2 Duo CPU-based computer using Microsoft Windows XP operating system. The sarcolemmal membrane charge balance equations, the *Ca*^2 + ^material balance equations in the myoplasm and SR, and the force balance equations describing the model for myofilament contraction constitute a set of 93 ordinary differential equations (ODEs). A fixed-step Merson-modified Runge-Kutta 4th-order numerical integration scheme [3] was used to solve this set of 1st-order differential equations (ODE) describing the dynamic model. The free *Ca*^2 + ^concentration in the dyad is governed by the time courses of the *Ca*^2 + ^ fluxes through *Ca*^2 + ^ transport systems, as well as by the time course of *Ca*^2 + ^binding to *Ca*^2 + ^ buffers present in the junction [4]. Description of the spatio-temporal dynamics of calcium transients in the dyad triggered by *Ca*^2 + ^stimulus (basis of CICR) requires calculation of the partial differential equations (PDE) of the whole reaction-diffusion system. Formation and dissipation of *Ca*^2 + ^gradients around an open channel (DHP-sensitive and Ry-sensitive channels in the dyad) is assumed instantaneous as was validated for microsecond timescale and nanoscopic space by Naraghi and Neher [5]. Local *Ca*^2 + ^concentration in the vicinity of open channels (located on opposing boundaries of the dyadic space) was calculated as the steady state gradient around a point source [6]. The *Ca*^2 + ^concentration increments from individual channels at each point in space were assumed to be additive [5,7]. The software kernel follows the changes in the state of trigger and release channels together with variables like membrane voltage and spatial *Ca*^2 + ^concentration to calculate the instantaneous rate constants and estimate the duration of transient events. Crank [8] discusses diffusion problems in a two-phase heterogeneous medium and shows that diffusion through a system of barriers (RyR feet structures in the dyadic cleft space) can be approximated by diffusion in the same region without barriers but with a reduced effective diffusion coefficient. We hence take this approach in modeling the *Ca*^2 + ^diffusion by solving the 2-D Laplacian equation (Krishna et al. [4], Appendix A3, Eq. 140) in the DCU without explicitly accounting for local potential fields. More specifically, an explicit finite difference scheme was used to solve these Laplacian equations describing *Ca*^2 + ^-diffusion in the dyadic space analogous to the method detailed in Smith et al. [9]. Specifically, a radial symmetry is employed in solving the PDE in the dyadic volume allowing the solution to be computed in a rectangular cross-section discretized into a 20 by 20 cartesian grid. The spatial step size used in the r and z-direction (Figure 1B, Krishna et al. [4]) was 10 nm and 0.76 nm respectively (Table two, Krishna et al. [4]). We use the method of lines (discretization in space) to solve the PDE. The full set of ODEs and finite difference equations are solved simultaneously to obtain the complete solution. Execution of a single cycle which translates to 200 ms at 5 Hz took 21 seconds with a time step of 1*μ*s. Results were visualized using Matlab (Mathworks, Natick, MA) and Origin (OriginLab Corp., Northampton, MA). 

## Model development

Our objective was to develop an integrated model of the rat ventricular cell under voltage clamp conditions, which includes the description of various *Ca*^2 + ^signalling pathways in the dyadic space, the myoplasmic medium and the sarcoplasmic reticulum (Figure 1, adopted from Krishna et al. [4]) as well as a comprehensive coupled mechanical system describing the contractile machinery responsible for force generation.

### Electrochemical description of ***Ca***^**2 +**^sub-system

Our model for the electrochemical description of the cell consists of an electrical-equivalent model for the cell membrane and a fluid-compartment model describing the flux of ionic species between the extracellular and several intracellular compartments (cell cytosol, SR and the dyadic coupling unit (DCU), in which resides the mechanistic basis of CICR). The DCU is described as a controller-actuator mechanism, internally stabilized by negative feedback control of the unit’s two diametrically-opposed *Ca*^2 + ^channels (trigger-channel and release-channel). It releases *Ca*^2 + ^ flux into the cytoplasm and is in turn enclosed within a negative feedback loop involving the SERCA pump, regulating *Ca*^2 + ^_*myo*_. A detailed description of the membrane classification, channel and exchanger distribution as well as the various fluid compartments involved is given in Krishna et al. [4]. Our model for the electrochemical description of the *Ca*^2 + ^ sub-system is based on our previous work [4] with the following modifications: (a) the rate constants used to model L-type *Ca*^2 + ^ current (*I*_*Ca,L*_), maximum *Na*^ + ^/*Ca*^2 + ^ exchanger current (*I*_*NaCa*_), maximum plasma membrane *Ca*^2 + ^-ATPase current (*I*_*PMCA*_) and the maximal uptake rate of the SERCA pump are Q_10_ adjusted using the values given in Table 1 to model temperature dependence; (b) The effect of cAMP-mediated *β*-adrenergic stimulation is modeled by allowing the relative regulatory activity of PKA to be a function of the available isoproterenol concentration (Appendix, Equations 57). 

**Table 1:** **Q**_**10**_** values used to model temperature variation**

**Parameter description**	**Q**_**10**_** value**	**References**
Rate constants modulating *I*_*Ca,L*_ channel kinetics	2.4	[10,11]
Maximum uptake rate of the SERCA pump	1.415	[12]
Maximum *Na*^ + ^/*Ca*^ + ^ exchange current (*V*_*max*_)	1.77	[12]
Maximum plasma membrane *Ca*^2 + ^-ATPase pump current (${\bar{I}}_{\text{PMCA}}$)	4.3	[12]

Q_10_ values in the electromechanical model were adopted from Rice et al. [13] with Qf_*app*_, Qh_*f*_, Qh_*b*_and Qg_*xb*_decreased to 2.25 to reproduce temperature dependence of peak force developed [14].

### Mechanical description of myofilament contractile system

**Figure 2:**
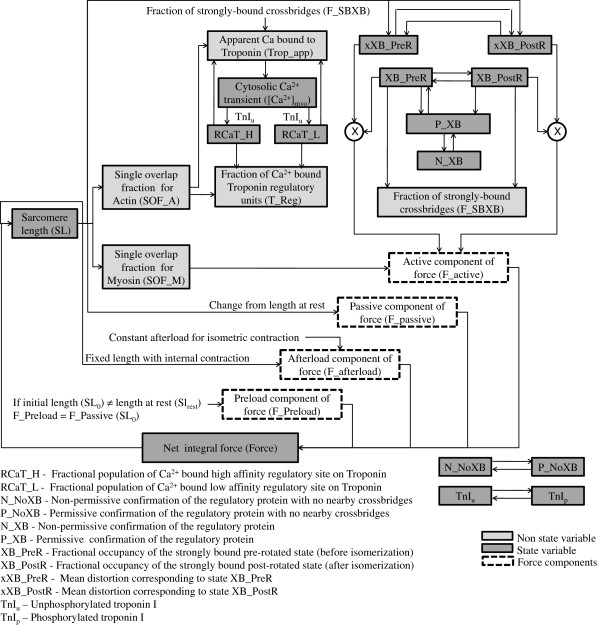
**Model for cardiac electro-mechanics.** Cooperative activation and crossbridge cycling. Model for cardiac electro-mechanics and force generation based on Rice et al. [13] shows states in non-permissive and permissive confirmations of the regulatory proteins. The permissive state transitions into a pre-rotated (XB_PreR) state having a strongly bound crossbridge with the head extended. The transition to the post-rotated (XB_PostR) force-generating state represents the isomerization to induce strain in the extensible neck region. Activation is triggered by the fraction of *Ca*^2 + ^bound troponin regulatory units (T_Reg) which sets the rate constant for transition between the non-permissive (N_XB) to permissive (P_XB) confirmation of the regulatory protein using a strong nonlinearity function to indicate cooperativity. The model assumes that troponin for regulation has affinity set by the thin filament overlap. The affinity for apparent *Ca*^2 + ^binding (used to perturb *Ca*^2 + ^_*myo*_), not only depends on thin filament overlap but also increases as crossbridges strongly bind to populate the pre-rotated and post-rotated states. The regulatory and apparent *Ca*^2 + ^binding terms are calculated separately to avoid a global feedback from strongly-bound crossbridges to *Ca*^2 + ^binding causing nonphysiological *Ca*^2 + ^sensitivity [13].

Our model for cardiac contractile mechanics (Figure 2) is based on the approximate model of cooperative activation and crossbridge cycling reported by Rice et al. [13] with the following modifications: (a) the first-order rate constants for the transformation of the troponin/tropomyosin regulatory complex (outside the single overlap region between the thick and thin filaments) from a crossbridge non-permitting state to a crossbridge permitting state and vice-versa are chosen as 500 s-1 and 50 s-1 respectively in order to reproduce results reported by Rice et al. [13]; (b) the *β*-adrenergic agonist isoproterenol (ISO) is known to cause a decrease in myofilament *Ca*^2 + ^sensitivity as a result of protein kinase A (PKA) mediated phosphorylation of troponin I [15,16] at Ser23/Ser24. Specifically, a two-state Markovian model is added to allow isoproterenol-dependent PKA-mediated interaction between troponin I (TnI) and the *Ca*^2 + ^-binding regulatory site on troponin. As shown in Figure 2, state TnI_*u*_, which denotes the unphosphorylated form of TnI, modulates the *Ca*^2 + ^ affinity of the regulatory site on troponin. This is in agreement with the findings of Messer et al. [17]. We model the effects of ISO by allowing the cumulative activation rate constant for *Ca*^2 + ^-binding to the troponin regulatory site to be a function of unphosphorylated TnI (TnI_*u*_), the availability of which is in turn dependent on the amount of ISO present (Appendix, Equations 15); (c) the large Q_10_ values used by Rice et al. (Qf_*app*_, Qh_*f*_, Qh_*b*_ and Qg_*xb*_, Table 1, [13]) are decreased from 6.25 to 2.25 in order to reproduce temperature dependence of peak force developed in intact thin rat ventricular trabeculae [14]. Although a calmodulin (CaM) mediated pathway has been reported [18] to be responsible for modulation of myofibrillar *Ca*^2 + ^-sensitivity (implying a possible CaM mediated role for Ca-dependent kinases or phosphatases in regulating myofilament contractility, particularly in frequency dependent acceleration of relaxation), we refrain from modeling this effect as the molecular mechanisms involved remain unresolved. 

### Testing protocol

We employ our coupled multiphysics model describing the electrochemical as well as the mechanical subsystems to study cellular contraction emphasizing the various modulatory influences that are at play. We begin with a detailed analysis of different types of twitch response to better understand the influence of various factors such as sarcomere length, peak *Ca*^2 + ^_*myo*_ and the stiffness of the contractile element in cell shortening, followed by a comparative study of these twitch responses. The negative feedback of cellular contraction on the myoplasmic *Ca*^2 + ^-transient [19,20] is also investigated. We then perform an idealized virtual experiment similar to that carried out in an experimental study [21] to uncover the regulation of cell contraction by *Na*^ + ^/*Ca*^2 + ^exchange, and in the process identifying the role of the SERCA pump in facilitating this effect. We then model the effects of temperature [22] on cardiac contractile response. This is followed by a study identifying the role of myofilament *Ca*^2 + ^sensitivity as a key factor influencing the degree of cell shortening. In particular, the effect of *β*-adrenergic agonist isoproterenol, which is known to cause a decrease in myofilament *Ca*^2 + ^sensitivity [15], is investigated. Hence, we have developed an integrated multiphysics model of rat ventricular cell electromechanics and now seek to study its response to various tests prescribed by elements of the virtual protocol above. In doing so, we hope to identify and clarify the role played by key factors involved in modulation of the cell’s contractile response.

## Results

From our modeling standpoint, the dyadic coupling unit (DCU) as defined by Krishna et al. [4] is a fundamental element involved in the mechanism of CICR. This previous study described the control features of this unit, as well as its interaction with the SERCA pump and free sarcolemmal pumps and exchangers to achieve a homeostatic regulation of myoplasmic *Ca*^2 + ^concentration. We now extend our voltage clamp studies to address the subject of force generation following CICR, starting with the classical twitch responses below.

### Twitch responses

A twitch response, which is a brief contractile response of a cardiac cell elicited by dynamically changing activator *Ca*^2 + ^, is a commonly used experimental characterization. Following are the three types of recordings commonly used to quantify force generation in isolated cardiac cells: (1) isosarcometric contraction which is experimentally obtained by incorporating feedback sarcomere length (SL) control using laser diffraction techniques [23-25]; (2) unloaded contraction where the cell is allowed to contract freely; and (3) isometric contraction where overall muscle length is fixed, but sarcomere length is not controlled, allowing considerable internal shortening as a result of compliant end connections (series elastic element). All the twitch studies are carried out at 22.5°C [13] to be consistent with the data cited. Under all three loading conditions, the cell is subjected to conditioning train of steady state (100 cycles) voltage clamp pulses at 5 Hz followed by a 0.8 s rest interval (allowing decay to a zero force resting state) which is subsequently followed by a single test twitch for which a transient lasting 1 s is obtained. The voltage clamp protocol used is a 50 ms step pulse to 10 mv from a holding potential of -40 mv. In this study, we report a normalized force with a maximum value of 1 possible under optimal conditions such as high *Ca*^2 + ^_*myo*_, isosarcometric loading (SL = 2.3 *μ*m) and low temperature allowing maximum overlap of thick and thin filaments.

#### Isosarcometric contraction

**Figure 3:**
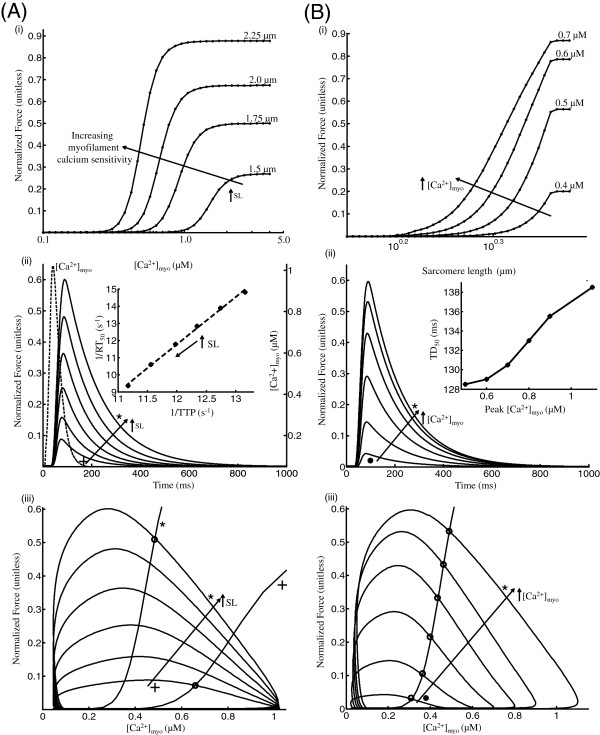
**Isosarcometric contraction.** Twitch response - Isosarcometric contraction (**A**) Modulation of sarcomere length - (i) Steady state F-Ca relationships for increasing SL. (ii) Traces for normalized force with SL varied from 1.8 (+) to 2.3 (∗) *μ*m in increments of 0.1 *μ*m. The *Ca*^2 + ^transient responsible for each of the traces is shown in the overlay. The inset shows the rate of relaxation versus rate of contraction for increasing sarcomere length (reciprocal of RT_50_, time taken for 50% sarcomere relaxation versus reciprocal of TTP, time taken for peak sarcomere contraction). (iii) Phase plots of self normalized force versus instantaneous [*Ca*^2 + ^]_*myo*_ for increasing SL overlayed with two steady state F-Ca relationships corresponding to SL = 1.8 (+) and 2.3 *μ*m (∗). (**B**) Modulation of Peak [*Ca*^2 + ^]_*myo*_ - (i) Steady state F-SL relationships for increasing background [*Ca*^2 + ^]_*myo*_. (ii) Sarcomere length is held constant at 2.3 *μ*m while the peak [*Ca*^2 + ^]_*myo*_ transient is scaled down by decreasing the voltage clamp pulse duration. The traces show the contractile response corresponding to myoplasmic *Ca*^2 + ^transients with peak values 1.1(∗), 0.9, 0.8, 0.7, 0.6, 0.5 (∙) *μ*M. The inset shows the relationship between TD_50_ (time taken from 50% activation to 50% relaxation) and activator *Ca*^2 + ^. (iii) Phase plots of self normalized force versus instantaneous [*Ca*^2 + ^]_*myo*_ for increasing peak [*Ca*^2 + ^]_*myo*_ overlayed with a steady state F-Ca relationships corresponding to SL = 2.3 *μ*m (∗). Model generated data corresponds to an idealized rat ventricular myocyte at 22.5°C.

**Figure 4:**
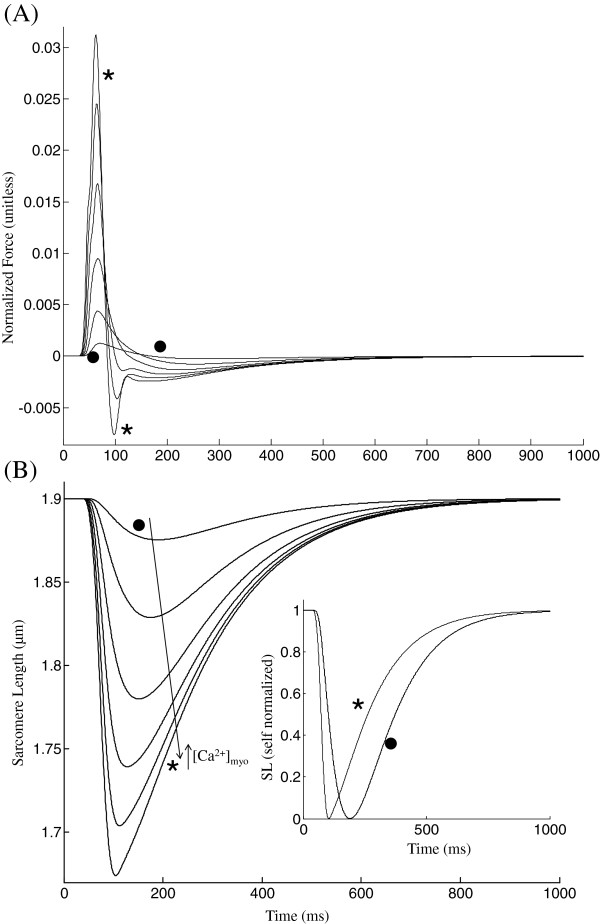
**Unloaded contraction.** Twitch response - Unloaded contraction. (**A**) The total muscle force is plotted corresponding to *Ca*^2 + ^transients with peak values 1.1 (∗), 0.9, 0.8, 0.7, 0.6, 0.5 (∙) *μ*M as in Figure 3B. (**B**) Cell shortening twitches as a function of *Ca*^2 + ^activation. The cell is allowed to contract from its equilibrium length of 1.9 *μ*m against the passive elastic and viscous restoring forces in the model of Rice et al. ([13]; Figure 1). Increasing peak translating into increased amount of activator *Ca*^2 + ^causes a decrease in time to peak shortening. The inset shows self-normalized sarcomere length for peak values of 1.1 (∗) and 0.5 (∙) *μ*M. Model generated data corresponds to an idealized rat ventricular myocyte at 22.5°C.

As mentioned above, in isosarcometric contraction, SL is maintained constant via external feedback control. We simulate this type of contraction under two distinct conditions: modulation of developed force by fixed changes in (A) SL, and (B) myoplasmic *Ca*^2 + ^concentration. In case ‘A’ while SL is fixed at different values, the input *Ca*^2 + ^-transient is kept identical (elicited by the standard 50 ms voltage clamp pulse at 5 Hz). In case ‘B’ while the level of activator *Ca*^2 + ^ is modulated, SL is kept fixed at 2.3 *μ*m.

(A) The steady state force-*Ca*^2 + ^ (F-Ca) relationship shown in Figure 3A-i exhibits a leftward shift and an increase in developed maximum plateau force as SL is clamped at increasing lengths. This leftward shift results from an increase in myofilament *Ca*^2 + ^sensitivity as SL is increased. Figure 3A-ii shows the temporal course of normalized force as SL is changed in steps from 1.8 to 2.3 *μ*m. The waveshape of standard *Ca*^2 + ^-transient is overlaid in dotted lines in this figure. Although an increase in SL (traces marked + to ∗) does not cause a large variation in the time to peak force (TTP), it does result in an increase in peak force magnitude and twitch duration as the result of an increase in myofilament *Ca*^2 + ^sensitivity. These characteristics show a strong correspondence with measured data from rat ventricular myocytes tested at similar (∼ 22.5°C) temperatures [14,24,26]. The correlation coefficient of the speed of contraction and relaxation has been experimentally observed [27] to be very close (> 0.98). The inset in Figure 3A-ii is a plot of the rate of relaxation (reciprocal of time taken for 50% sarcomere relaxation (RT_50_)) versus rate of contraction (reciprocal of time taken for peak sarcomere contraction (TTP)) for increasing SL. This linear relationship highlights contraction-relaxation coupling, and represents a key intrinsic property of the contractile myofilaments [27]. Figure 3A-iii shows the phase plots of self normalized force versus the instantaneous *Ca*^2 + ^concentration in the cytosol for increasing SL (traces marked + to ∗) overlayed with two steady state F-Ca relationships corresponding to SL = 1.8 *μ*m (+) and SL = 2.3 *μ*m (∗). The assessment of dynamic and steady-state *Ca*^2 + ^relationships allows better analysis of the phase-plane loops of force versus *Ca*^2 + ^. The active twitch curve is related to the steady-state values to determine, at what isochrone the dynamic force-*Ca*^2 + ^ value equals that obtained in the steady-state relationship. This point of intersection of the steady state F-Ca trace and the corresponding phase plot gives the contraction-relaxation coupling point (CRCP, marked as ∘) from initiation of stimulation [16]. Time is implicit on the phase trajectory and at time instants prior to reaching the critical coupling point for a particular trajectory, *Ca*^2 + ^_*myo*_ exceeds the value of *Ca*^2 + ^predicted by the steady state F-Ca relationship. This excess favors continued sarcomere contraction. At later time points beyond the CRCP, the developed force is greater than that predicted by the steady state curve, which favors myofilament relaxation. 

(B) Increasing background *Ca*^2 + ^_*myo*_ causes a leftward shift in steady state F-SL relationship as shown in Figure 3B-i. The increase in maximal plateau force with increase in background *Ca*^2 + ^_*myo*_ is observed to be less prominent at higher levels of activator *Ca*^2 + ^in the myoplasm. In Figure 3B-ii the activator *Ca*^2 + ^ is varied by modulating the peak of the *Ca*^2 + ^_*myo*_ transient by adjusting the voltage clamp pulse duration (an increase in pulse duration from 5 ms to 50 ms increased peak *Ca*^2 + ^_*myo*_ from 0.5 to 1.1 *μ*M respectively). This protocol allows for the peak of the transient to be changed without a significant change in the duration of the transient (Krishna et al. [4] ; Figure 4). The traces correspond to increasing peak values from 0.5 (∙) to 1.1 (∗) *μ*M. Although similar to case with increasing SL, increasing activator *Ca*^2 + ^ results in a relatively non-linear increase in peak force generated. As shown in Figure 3A-i, the steady state F-Ca relationship is characterized by a Hill function as experimentally observed [25]. The time-to-peak force (TTP) remains relatively unaffected by the amount of activator *Ca*^2 + ^causing the twitch response. Inset in Figure 3B-ii shows the dependence of TD_50_ (time taken from 50% activation to 50% relaxation) on peak *Ca*^2 + ^_*myo*_ indicating an increase in twitch duration with increasing levels of activator *Ca*^2 + ^. Figure 3B-iii shows the phase plots of self normalized force versus the instantaneous *Ca*^2 + ^concentration in the myoplasm for increasing peak *Ca*^2 + ^_*myo*_ (traces marked ∙ to ∗) overlayed with a steady state F-Ca relationships corresponding to SL = 2.3 *μ*m (∗). The contraction-relaxation coupling point (∘) traverses along the F-Ca relationship to increasing values of *Ca*^2 + ^ and force with increasing peak *Ca*^2 + ^_*myo*_. 

#### Unloaded contraction

**Figure 5:**
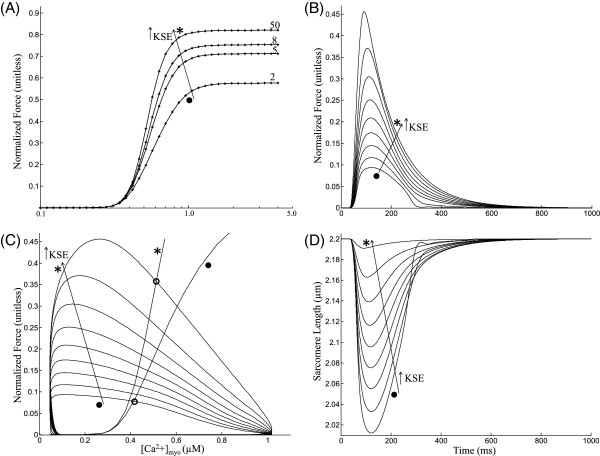
**Isometric contraction.** Twitch response - Isometric contraction. The cell is held at a constant total length but the sarcomere is allowed to contract via a series elastic element. (**A**) Steady state F-Ca relationships for increasing KSE values. (**B**) Traces for total muscle force during an isometric twitch with KSE values of 1(∙), 1.4, 2, 3, 4, 5, 7, 10 and 50 (∗) where units of KSE are normalized force-per-micrometer extension. (**C**) phase plots of self normalized force versus [*Ca*^2 + ^]_*myo*_ for increasing KSE values (corresponding to panel B) overlayed with two steady state F-Ca relationships corresponding to KSE = 1.0 (∙) and 50.0 (∗). (**D**) Sarcomere length traces showing internal shortening. Model generated data corresponds to an idealized rat ventricular myocyte at 22.5°C.

The protocol for the unloaded case is as follows. The cell is not stretched with pre-load so that the series elastic element is unattached and is therefore not in play. In the model of Rice et al. ([13]; Figure 1), the contractile element is shunted by elastic and viscous damping elements. In that figure, the nonlinear elastic element is characterized by a cubic force vs SL characteristic centered about an equilibrium point (SL_0_=1.9 *μ*m; F=0). In the unloaded case without stimulation, any stored energy in the system is dissipated and SL decays to the equilibrium point on the passive force vs SL characteristic. With electrical activation and subsequent *Ca*^2 + ^ release, active force is developed and SL shortening occurs against the aforementioned passive restoring forces. Providing the same sequence of voltage clamp pulses as in Figure 5, an identical sequence of *Ca*^2 + ^-transients is produced to drive the active contractile mechanism. Figure 5A is a plot of total developed force (active and passive) as a function of peak *Ca*^2 + ^_*myo*_. This net instantaneous force can become negative when the magnitude of the passive forces exceeds that of the active component (Figure 5A). Thus, an increase in activator *Ca*^2 + ^ causes an increase in peak force generated, which translates into enhanced shortening. Corresponding changes in sarcomeric length as shown in Figure 5B indicate that increasing levels of activator *Ca*^2 + ^result in a decrease in time to peak (TTP declined from 156.0 ms (∙) to 70.5 ms (∗)) and an increase in the rate of relaxation (RT_50_ computed from time of peak decreased from 183.0 ms (∙) to 157.5 ms (∗)). 

#### Isometric contraction

**Figure 6:**
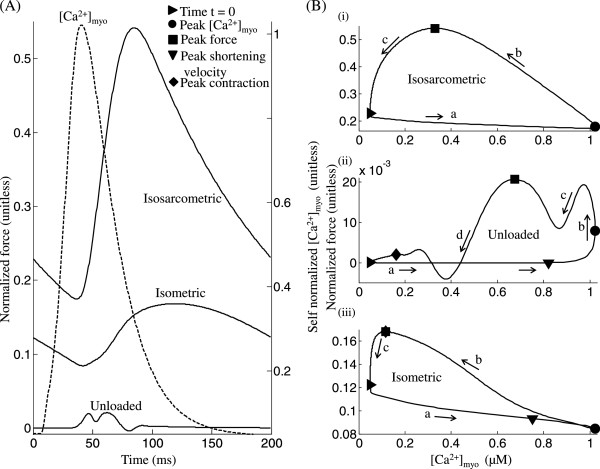
**Comparison of twitch responses.** Twitch responses - A comparison between three types of steady state twitch responses from an isolated rat ventricular myocyte viz. isosarcometric, unloaded and isometric (KSE = 2). Fixed SL in the isosarcometric case and the initial pre-contraction SL in the isometric case are chosen as 2.2 *μ*m. Equilibrium SL in the unloaded case is 1.9 *μ*m. (**A**) Traces for normalized force in each of the three cases with an overlay of normalized [*Ca*^2 + ^]_*myo*_ transient responsible for the twitch and (**B**) Phase plots of normalized force versus the instantaneous *Ca*^2 + ^concentration in the cytosol in each of the three cases. Note the overlap of ♦ and ■ in panel iii. Model generated data corresponds to an idealized rat ventricular myocyte at 22.5°C.

A third type of twitch can be simulated where the cell is kept at a fixed total length, allowing it to contract in response to *Ca*^2 + ^release by internal shortening of the sarcomere made possible by a non-contractile series elastic element whose stiffness (KSE value) dictates the end compliance and hence the degree of internal shortening. Increasing KSE values causes an increase in maximal plateau force in steady state F-Ca relationship as shown in Figure 6A. Figure 6B shows traces for total force (sum of both passive and active force) during an isometric twitch corresponding to KSE values increased from 1.0 to 50.0 normalized force-*μ*m^−1^. With an increase in end compliance (decrease in KSE), the degree of internal shortening increases and the total force measured at the cell end decreases, showing a delayed peak and an increase in rate of relaxation (Figure 6B). The delayed peak occurs because the peak force is measured when the series elastic element is at its maximum length, which occurs with greater delay with increasing end compliance. Increasing end compliance decreases twitch duration (Figure 6B) because, as observed experimentally [28] re-lengthening hastens relaxation as a result of an increase in mean distortion of the strongly bound crossbridge states (xXB_PreR, xXB_PostR in Figure 2) which causes a decrease in the forward rotation rate of the crossbridges (Eqn. 22, Rice et al. [13]) and hence a faster force decline. Figure 6C shows the phase plots of self normalized force versus the instantaneous *Ca*^2 + ^concentration in the myoplasm for increasing KSE values (traces marked ∙ to ∗) overlayed with two steady state F-Ca relationships corresponding to KSE = 1.0 (∙) and KSE = 50.0 (∗). The contraction-relaxation coupling point (∘) moves to increasing values of *Ca*^2 + ^ and force with increasing KSE values with the relative change in *Ca*^2 + ^ being smaller than force. Figure 6D shows the corresponding traces for sarcomere length during the isometric twitch. As the KSE value is increased from 1 to 50 the decreasing compliance results in a decline in cell shortening accompanied by a decrease in time to peak shortening from 122 ms to 92 ms (Figure 6D). 

**Figure 7:**
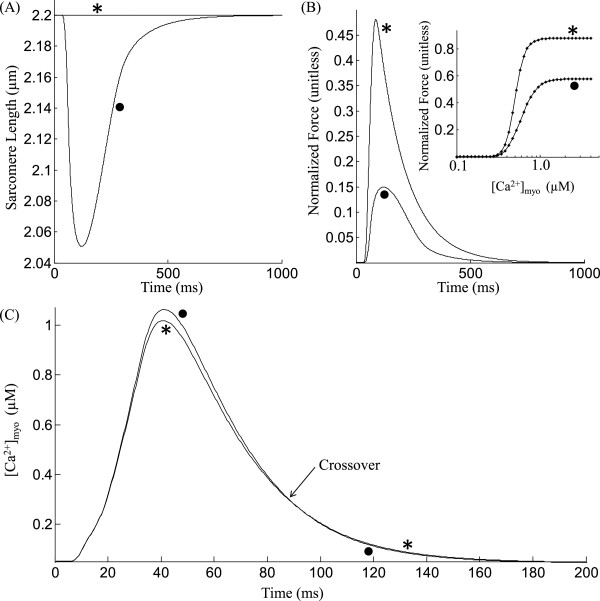
**Feedback length effects.** Feedback of internal shortening on myoplasmic [*Ca*^2 + ^]_*myo*_ transient. The protocol used comprises of 9 beats of isometric contraction followed by a 0.8 s rest interval and beat 10 (shown above) when the cell is allowed to internally shorten (∙) or held at a fixed sarcomere length of 2.2 *μ*m (∗). (**A**) The sarcomere length in both the cases shows the degree of contraction when the cell is allowed to internally shorten via a series elastic element (KSE = 2). (**B**) Isosarcometric case shows enhanced force when compared to the isometric case. The inset shows corresponding steady state F-Ca relationships (**C**) As seen in experimental studies, the isosarcometric case shows a modest decrease in [*Ca*^2 + ^]_*myo*_ transient. Model generated data corresponds to an idealized rat ventricular myocyte at 22.5°C.

Figure 7 shows the three types of simulated twitch responses studied, compared in their force vs. time plots, as well as in their normalized force vs. *Ca*^2 + ^_*myo*_ phase diagrams. This plotting format aids in drawing a comparison that highlights the unique characteristics of each loading condition. The protocol used here is a steady state 5 Hz stimulation without a rest interval before the test twitch (unlike Figures 3, 5 and 6). Figure 7A shows that the isosarcometric case results in maximum force development, whereas the unloaded case records the minimum force for identical sarcomere length and initial conditions. Figure 7B shows the phase plots of normalized force versus the instantaneous *Ca*^2 + ^concentration in the cytosol constructed from model-generated data captured at steady state (the last in a train of stimuli comprising 100 cycles at 5 Hz stimulation) from a twitch caused by a *Ca*^2 + ^_*myo*_ transient resulting from a voltage clamp pulse (amplitude -40 mv to 10 mv and a duration of 50 ms). The initial pre-contraction sarcomere length in the isometric case and the sarcomere length clamp in the isosarcometric case are both set to 2.2 *μ*m whereas the equilibrium length in the unloaded case is chosen as 1.9 *μ*m [13]. 

In the case of an isosarcometric contraction the net force comprises only the active component due to the tension generated by the sarcomere trying to contract. Phase ‘a’ in Figure 7B-i is indicative of the delay in the contractile response when compared to the [*Ca*^2 + ^]_*myo*_ transient. However, soon after the [*Ca*^2 + ^]_*myo*_ reaches its peak (∙), the sarcomere begins to contract, resulting in a gradual increase in force, achieving a maximum (■) as seen in phase ‘b’ of Figure 7B-i. As active force increases, a decline in level of activator *Ca*^2 + ^in the cytosol ultimately causes a recovery to the minimum contractile state (▸), as shown in phase ‘c’ of Figure 7B-i.

Total force generated by an unloaded cell during contraction against its internal restoring force is a combination of the active component attributed to tension generating action of cycling crossbridges and the passive component attributed to titin and other cytoskeletal elements. Passive force generated has a negative contribution to the net force for SL values lower than the equilibrium length. Hence, the competition between the active and passive components of force gives the trace in Figure 7B-ii its characteristics. During phase ‘a’ in Figure 7B-ii, as the *Ca*^2 + ^level in the cytosol increases towards its peak value (∙), the sarcomere attains maximum contraction velocity (▾), which is followed by a steep increase in net force as shown in phase ‘b’ of Figure 7B-ii. However, decreasing sarcomere length increases the negative contribution from the passive component of force resulting in the first transient decline shown in phase ‘c’ of Figure 7B-ii. The increased *Ca*^2 + ^binding to troponin in response to the rise in activator *Ca*^2 + ^ in the cytosol enhances the active component of force, allowing the cell to reach peak net contractile force (■). The fast decline in sarcomere length which causes a rapid increase in the passive component of force results in the second transient decline as shown in phase ‘d’ of Figure 7B-ii. However, a subsequent decrease in cell shortening soon allows an increase in net force lasting for a short duration (while the SL reaches its minimum (♦)). The declining *Ca*^2 + ^level in the cytosol, an outcome of SR uptake, then causes a gradual recovery to the resting state (▸).

As shown in Figure 7A, for the same activator *Ca*^2 + ^, although the total muscle force generated during isometric contraction exhibits a triphasic response similar to an isosarcometric contraction, its own unique characteristics are a delayed time to peak (an increase from 84.5 ms to 119 ms) and relatively smaller magnitude. As shown in phase ‘a’ of Figure 7B-iii, the increase in *Ca*^2 + ^concentration is not reflected in a fast mechanical response. After [*Ca*^2 + ^]_*myo*_ reaches its peak (∙), the tension in the sarcomere begins to build up (phase ‘b’ of Figure 7B-iii) although at a slower rate (compare isometric and isosarcometric traces in Figure 7A), causing a delayed time to peak (84 ms and 120 ms in isosarcometric and isometric cases, respectively) due to the presence of the series elastic element which facilitates slow internal shortening. The sarcomere achieves peak contraction (♦) when the total muscle force reaches its maximum (■), following which the cell recovers back to the minimum contractile state (▸) as shown in phase ‘c’, Figure 7B-iii. During isometric contraction (KSE = 2) the afterload (due to the series elastic element) tracks the active component of force generated due to the tension developed in the sarcomere while the passive component of force (attributed to titin and other cytoskeletal elements) is small in magnitude owing to a much smaller degree of sarcomere contraction achieved when compared to the unloaded case (compare trace marked ∗ in Figure 5B and the trace for KSE=2 in Figure 5D).

### Effect of contraction on the [***Ca***^**2 +**^]_*myo*_ transient

**Figure 8:**
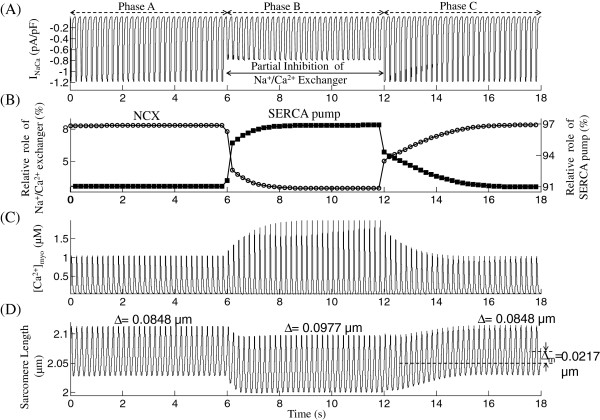
**Regulation of contraction via NCX.** Regulation of isometric cell contraction by *Na*^ + ^/*Ca*^2 + ^exchange. The protocol used for the isometric contraction is stimulation at 5 Hz comprising of 30 beats when the cell undergoes unperturbed contraction (phase A) followed by 30 beats (phase B) when the *Na*^ + ^/*Ca*^2 + ^exchanger function is partially inhibited after which the inhibition is relieved allowing the cell to recover to a steady state control during the following 30 beats (phase C). (**A**) The *Na*^ + ^/*Ca*^2 + ^exchanger current (*I*_*NaCa*_) showed a rapid transition between the control and inhibited phase and vice versa. (**B**) Relative role of the *Na*^ + ^/*Ca*^2 + ^exchanger and the SERCA pump. (**C**) Myoplasmic *Ca*^2 + ^concentration. (**D**) Sarcomere length in response to *I*_*NaCa*_inhibition. Model generated data corresponds to an idealized rat ventricular myocyte at 22.5°C.

The myoplasmic *Ca*^2 + ^_*myo*_ transient which follows SR release acts as the trigger for myofilament contraction. However, the contracting myofilament also has a feedback effect on the shape of the *Ca*^2 + ^_*myo*_ transient as a result of *Ca*^2 + ^binding to the low affinity regulatory sites on troponin in the myofilament. The *Ca*^2 + ^affinity of this site depends on both the sarcomere length as well as the fraction of strongly-bound crossbridges (Eq. 37, Rice et al. [13]). The protocol used to test these effects is similar to that employed by Janssen and de Tombe [29], wherein the cell is stimulated at 5 Hz for 9 beats under conditions of isometric contraction (KSE=2) followed by a 0.8 s rest interval allowing decay to a zero force resting state. This is followed by the 10th beat for which a transient lasting 1 s is obtained. On the 10th beat, the cell is allowed to sarcometrically shorten as usual (∙) or a sarcometric length clamp is imposed at a fixed sarcomere length of 2.2 *μ*m (∗). At the onset of *Ca*^2 + ^-activation of troponin on the 10th beat, initial conditions for the two different loading tests (isometric and isosarcometric) are identical. Sarcomere length changes under both loading conditions are shown in Figure 8A. As observed earlier in Figures 7B (i) and (iii) as well as in Figure 8B, isosarcometric conditions generate a larger force than isometric, due to enhanced myofilament *Ca*^2 + ^sensitivity reflected by a decrease in EC_50_ from 0.59 *μ*M (∙) to 0.5 *μ*M (∗) (steady state F-Ca relationships shown in the inset in Figure 8B) and increased *Ca*^2 + ^-binding to troponin. This binding causes a small (<1%) decline in the magnitude of the *Ca*^2 + ^_*myo*_ transient (Figure 8C). Subsequently, as the *Ca*^2 + ^-transient starts to decay, *Ca*^2 + ^dissociates from troponin, slightly decreasing the rate of decline of the *Ca*^2 + ^-transient. When the falling phases of the *Ca*^2 + ^-transients for the two loading conditions are compared, the isosarcometric *Ca*^2 + ^-transient decays more slowly and its curve crosses over the isometric *Ca*^2 + ^-transient. Although Janssen et al. [29] reported an increase in *Ca*^2 + ^transient due to uncontrolled shortening, the crossover was not observed as the noise level was too large. However, the crossover effect has been reported in other studies on rat ventricular trabeculae [19,20] using long and short sarcomere length twitches producing larger changes in developed force and hence the shape of the *Ca*^2 + ^_*myo*_ transient (>5% change in peak value). 

### Regulation of isometric cell shortening by ***Na***^** +**^**/*Ca***^**2 +**^exchange

**Figure 9:**
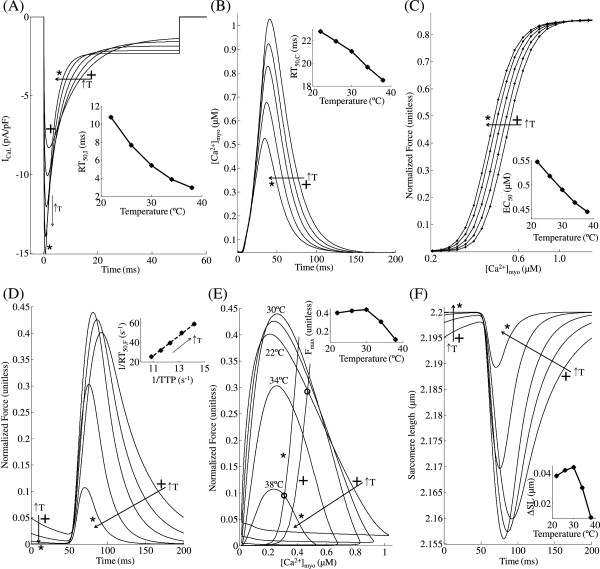
**Temperature dependence of twitch response.** Isometric twitch response (KSE = 10.0) for temperatures ranging from 22°C to 38°C at 5 Hz stimulation. (**A**) Traces for L-type *Ca*^2 + ^current (*I*_*Ca,L*_) showing increasing peak and rate of decline with increase in temperature. The inset shows the temperature dependence of RT_50,*I*_, time required for 50% *I*_*Ca,L*_inactivation. (**B**) Traces for [*Ca*^2 + ^]_*myo*_ transient for increasing temperatures show a decline in peak. The inset shows the temperature dependence of RT_50,*C*_, time required for 50% decline in [*Ca*^2 + ^]_*myo*_ from its peak value. (**C**) Steady state F-Ca relationships for increasing temperature. The inset shows the temperature dependence of half maximal effective concentration (EC_50_). (**D**) Traces showing temperature dependence of normalized isometric force developed. The inset shows the relationship between rate of relaxation and rate of contraction. (**E**) Phase plots of normalized force versus instantaneous [*Ca*^2 + ^]_*myo*_ for increasing temperature overlayed with two steady state F-Ca relationships corresponding to 22°C (+) and 38°C (∗). The inset shows temperature dependence of peak force developed. (**F**) Sarcomere length corresponding to traces for force developed in panel D.

During stable, steady-state operation, *Ca*^2 + ^ entry into the cytosol via *I*_*Ca,L*_ and SR release must exactly balance *Ca*^2 + ^ efflux via the sarcolemmal *Na*^ + ^/*Ca*^2 + ^ exchanger (NCX), plasma membrane *Ca*^2 + ^-ATPase pump, and *Ca*^2 + ^-uptake to the SR by the SERCA pump. To study the role of the NCX in isometric SL shortening we develop a virtual experimental protocol loosely patterned after a study on rat ventricular myocytes [21]. In our experiment, isometric contraction is stimulated by voltage clamp pulses at 5 Hz. In phase A of the experiment, 30 clamp pulses are applied resulting in regular isometric contractions. This is immediately followed by phase B where the NCX is assumed partially inhibited (achieved in the model by a 25% decrease in maximum *Na*^ + ^/*Ca*^2 + ^exchange current) by rapid superfusion of a bathing solution containing a NCX inhibitor (e.g. Phe-Arg-Cys-Arg-Ser-Phe-CONH2 (FRCRSFa) or exchanger inhibitory peptide (XIP) which are known [30] to cause selective NCX inhibition). This is followed by phase C where rapid superfusion with normal bathing solution completely removes the inhibitor, thus restoring exchanger activity to initial levels. It is well known that a decrease in NCX activity (Phase B, Figure 9A) results in an increase in SR *Ca*^2 + ^ content as a result of the excess *Ca*^2 + ^in the cytosol being resequestered into the SR via the SERCA pump. Figure 9B elucidates the relative contribution of the *Na*^ + ^/*Ca*^2 + ^ exchanger and the SERCA pump in *Ca*^2 + ^extrusion from the cytosol, showing an increase in the relative role of SERCA pump as a result of inhibition in NCX activity. As observed experimentally [21] this enhancement in SR *Ca*^2 + ^content results in an increased availability of activator *Ca*^2 + ^following release (Figure 9C) which in turn enables improved cell shortening as seen by an enhancement in peak to peak amplitude of contraction in Figure 9D (*Δ* increases from 0.0848 *μ*m to 0.0977 *μ*m) and is accompanied by a decrease in mean sarcomere length (*Δ*_*m*_ = 0.0217 *μ*m). A similar study (not shown here) involving a sudden increase in NCX activity instead of a decrease resulted in an opposite (a decrease in SR *Ca*^2 + ^ content causing diminished release and hence a decrease in degree of contraction) symmetric response of similar magnitude indicating strong homeostatic control. Myoplasmic *Na*^ + ^concentration (17 mM) remains relatively constant throughout and hence is not involved in this indirect control of myofilament contractility. Our virtual experiment only approximates the actual experiment in that we assume the ability to make very rapid changes in the composition of the bathing medium relative to the time constants involved in the response. However, the model generated responses in steady state can be compared with measured steady state response in the presence/absence of NCX inhibition using selective blocking agents that allow complete post-washout recovery [30]. 

### Effect of temperature on contractile performance

**Figure 10:**
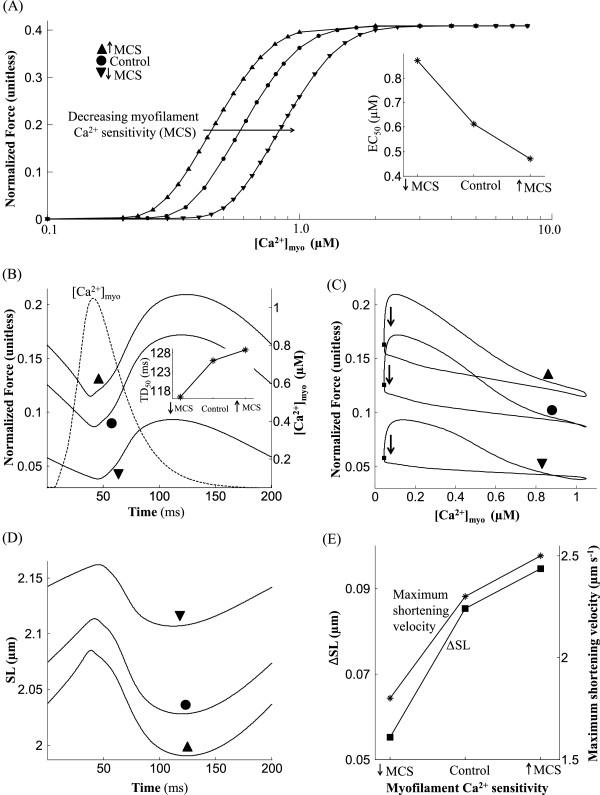
**Role of myofilament *Ca***^***2 +***^** sensitivity.** Dependence of isometric contraction on myofilament *Ca*^2 + ^sensitivity (MCS). (**A**) The steady state normalized force versus [*Ca*^2 + ^]_*myo*_ relationship shows a rightward shift with decreasing myofilament *Ca*^2 + ^sensitivity. (**B**) Traces for normalized force recorded at steady state with an overlay of the [*Ca*^2 + ^]_*myo*_ transient. The inset shows MCS dependent changes in TD_50_ (time taken from 50% activation to 50% relaxation). (**C**) Phase plots of normalized force versus the instantaneous *Ca*^2 + ^concentration in the cytosol. (**D**) Traces for sarcomere length indicating increased shortening with temperature. (**E**) Degree of sarcomere shortening and peak shortening velocity as a function of MCS. Model generated data corresponds to an idealized rat ventricular myocyte at 22.5°C driven by standard 50 ms voltage pulses at a repetition frequency of 5 Hz.

Temperature is known to have a strong effect on the L-type *Ca*^2 + ^ current (*I*_*Ca,L*_), the *Ca*^2 + ^-transient and the contractile mechanics. One very significant effect of temperature on whole-cell *I*_*Ca,L*_ is the pronounced increase in its rate of decline with an increase in temperature [31,32]. Thus, with an increase from room to body temperature, peak inward trigger current increases but the waveform becomes much narrower. Figure 10A shows model-generated *I*_*Ca,L*_waveforms at temperatures between 22°C (+) and 38°C (∗) in steps of 4°C, where one can observe the increase in peak current but also the increased rate of decline in the trigger current waveform with an increase in temperature. Specifically, peak *I*_*Ca,L*_ at 22°C was 8.31 pA/pF compared with 15.05 pA/pF at 38°C, whereas time taken for 50% 50% *I*_*Ca,L*_ inactivation (RT_50,*I*_) decreased from 10.75 ms at 22°C to 2.95 ms 38°C (inset in Figure 10A). These indices are in general agreement with measured voltage clamp data [32,33] obtained from rat ventricular trabeculae. 

Although skinned rat ventricular preparations have been used extensively in studies of the cardiac contractile process, data from such preparations violates the assumptions of our whole cell electromechanical model. The integrity of the plasma membrane components and intricate *Ca*^2 + ^-regulatory system are compromised to some degree, regardless of the skinning technique used. Therefore, we have chosen to consider only data from rat ventricular myocytes or ultra-thin rat ventricular trabeculae to help validate the model.

The decrease in integrated *I*_*Ca,L*_and faster SR uptake with increasing temperature together cause a decline in peak and duration of the *Ca*^2 + ^-transient as shown in Figure 10B. This agrees with the experimental findings by Janssen et al. ([14]; Figure 1) on thin rat ventricular trabeculae. RT_50,*C*_ corresponding to the *Ca*^2 + ^-transient (time taken for 50% decline in *Ca*^2 + ^_*myo*_) decreased from 22.8 ms at 22°C to 18.5 ms at 38°C (inset in Figure 10B). Myofilament *Ca*^2 + ^sensitivity increases with an increase in temperature as a result of a temperature dependent enhancement in crossbridge cycling rate [34-36]. Traces for the steady state F-Ca relationship in Figure 10C show a temperature dependent increase in myofilament *Ca*^2 + ^ sensitivity with no significant change in maximum plateau force. An increase in temperature from 22°C (+) to 38°C (∗) results in a decrease in EC_50_ from 0.55 *μ*M to 0.44 *μ*M (inset in Figure 10C).

As experimentally observed [14], a decrease in peak and duration of the *Ca*^2 + ^_*myo*_-transient with increasing temperature results in a corresponding overall decrease in peak developed contractile force and twitch duration. Our simulations show changes in *I*_*Ca,L*_ and *Ca*^2 + ^_*myo*_ in Figures 9A and B, and corresponding changes in developed force in Figure 10D with increasing temperature. As the temperature is increased from 22°C to 30°C, traces for normalized force in Figure 10D show a small increase in peak amplitude despite a decline in the amount of activator *Ca*^2 + ^responsible for contraction due to a temperature dependent increase in myofilament *Ca*^2 + ^sensitivity (Figure 10C). This increase in peak developed force at low, non-physiological temperatures is confirmed by Janssen et al. ([14]; Figure 1). However, as the temperature is increased further from 30°C to 38°C, further decreases in peak *Ca*^2 + ^_*myo*_ cause strong decreases in peak developed force. Figure 10D shows that an increase in temperature was accompanied by a decrease in both time to peak (TTP) as well as RT_50,*F*_ (time taken for 50% decline in force). The inset in Figure 10D shows the linear relationship [27,37] between rate of relaxation versus rate of contraction for increasing temperature (reciprocal of RT_50,*F*_versus reciprocal of TTP).

Figure 10E shows phase plots of normalized force versus instantaneous *Ca*^2 + ^concentration in the myoplasm for increasing temperatures (22°C (+) to 38°C (∗) in steps of 4°C) overlayed with two steady state F-Ca relationships corresponding to 22°C (+) and 38°C (∗). The contraction-relaxation coupling point (∘) moves to decreasing values of [*Ca*^2 + ^] and force with increasing temperature. Phase-plane analysis of normalized force versus instantaneous *Ca*^2 + ^concentration in the myoplasm reveals that, as the temperature is increased from 22°C to 38°C, the relaxation phase moves to the right towards the corresponding steady-state F-Ca relationship. This suggests that, with increase in temperature there is a departure from cross-bridge kinetics being the rate-limiting step in cardiac relaxation to a more *Ca*^2 + ^-driven mechanism. As evident from Figure 10D, the inset in Figure 10E shows the temperature dependence of peak force developed indicating a moderate increase at low (< 30°C) temperatures with a strong decline at temperatures above 30°C. Traces for SL shortening in Figure 10F corresponding to [*Ca*^2 + ^]_*myo*_ transients in panel B, indicate an overall decrease in sarcomere shortening with increasing temperature which agrees with the trend in developed force in panel D. Comparison of insets in Figure 10E and F shows the correlation between peak force developed and the corresponding delta change in SL. The effect of change in temperature on myofilament contractility is a two-stage response with feedback. Firstly, the [*Ca*^2 + ^]_*myo*_ transient is temperature sensitive owing largely to the temperature dependence of the trigger current *I*_*Ca,L*_. Secondly, the cellular contractile machinery is highly temperature sensitive due the strong temperature dependence of rate kinetics involved in the formation of crossbridges. In addition, the process of crossbridge formation is known to have a small feedback effect on the [*Ca*^2 + ^]_*myo*_ transient as seen in Figure 8. As the temperature is increased from 22°C to 30°C, the temperature sensitivity of crossbridge kinetics predominates the temperature dependence of [*Ca*^2 + ^]_*myo*_ transient in determining the contractile response. However, for temperatures from 30°C to 38°C the opposite holds true.

### Role of myofilament ***Ca***^**2 +**^sensitivity

**Figure 11:**
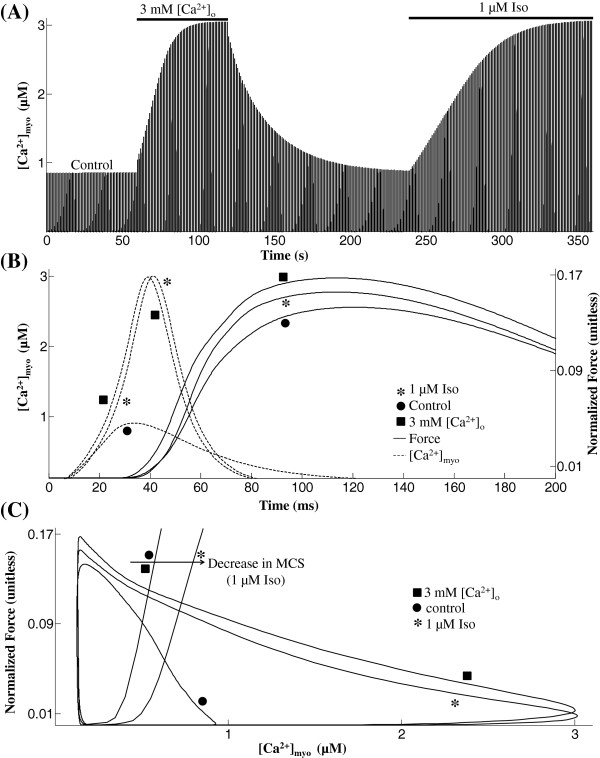
**Effect of isoproterenol and increase in [*Ca***^***2 +***^]_***o***_**.** Role of isoproterenol and [*Ca*^2 + ^]_*o*_. (**A**) Time record of [*Ca*^2 + ^]_*myo*_ transient in the control case as well as with 3 mM [*Ca*^2 + ^]_*o*_ and 1 *μ*M ISO. (**B**) Traces for normalized force recorded at steady state with an overlay of [*Ca*^2 + ^]_*myo*_ transient. (**C**) Phase plots of normalized force versus the instantaneous *Ca*^2 + ^concentration in the cytosol with an overlay of traces showing normalized steady state force versus [*Ca*^2 + ^]_*myo*_ relationship. Model generated data corresponds to an idealized rat ventricular myocyte at 22.5°C.

Among various factors influencing the degree of cell shortening achieved in response to myoplasmic *Ca*^2 + ^_*myo*_ transient, the *Ca*^2 + ^affinity of troponin C regulatory site is known to be particularly important. Here, we studied the dependence of isometric force response on myofilament *Ca*^2 + ^ sensitivity (MCS). An increase in *Ca*^2 + ^ affinity is modeled by a 30% increase in *k*_*onT*_ (traces marked ▴ in Figure 11), the rate constant for *Ca*^2 + ^-binding to troponin regulatory site. Similarly, a decrease in MCS is modeled by a corresponding decrease in *k*_*onT*_ (traces marked ▾ in Figure 11). The steady state F-Ca relationships in Figure 11A show the gradual rightward shift in response to decreasing MCS as seen experimentally in right ventricular trabeculae from rat in the presence of bupivacaine [38]. A positive/negative change in MCS results in the average *Ca*^2 + ^_*myo*_ required for half-maximal activation to decrease/increase from 0.61 *μ*M in the control case to 0.47 *μ*M and 0.89 *μ*M respectively. However, the Hill coefficient was constant (4.0) showing no change in response to modulation in MCS. 

Figure 11B shows traces of normalized force captured at steady state in response to a [*Ca*^2 + ^]_*myo*_ transient (overlaid) in the control case as well as with modified myofilament *Ca*^2 + ^sensitivity. A 30% decrease (from control value) in MCS causes a much larger change in peak isometric force generated than a 30% increase, highlighting the nonlinear response. Figure 11B shows that an increase in MCS causes a faster onset of the upstroke in force response and a delay in the start of recovery as a result of enhancement in *Ca*^2 + ^ binding to troponin. This delay in recovery manifests in an increase in time to peak from 119.5 ms (∙) in the control case to 124 ms (▴). Similarly, a decrease in MCS produces an opposite effect resulting in a decrease in time to peak (112 ms (▾)). The two distinct slopes during the upstroke in force response in Figure 11B are a result of an initial contribution of the strongly bound pre-rotated state (XB_PreR in Figure 2) followed by the effect of increase in strongly bound post-rotated state (XB_PostR in Figure 2). As shown in the inset in Figure 11B, an increase in MCS causes an increase in TD_50_ (time taken from 50% activation to 50% relaxation).

Phase plots of normalized force versus the instantaneous *Ca*^2 + ^ concentration in the cytosol are shown in Figure 11C. As observed experimentally [39], a decrease in myofilament *Ca*^2 + ^sensitivity causes the gradient (units of Normalized force/*μ*M) of the trajectory during the relaxation phase of the twitch contraction (marked by thick arrows in Figure 11C) to decrease from 12.4 in the control case to 7.0. A similar but opposite effect was observed in the rate of relaxation with a corresponding increase in MCS. A delayed onset of the upstroke in force response as a result of a decrease in MCS (Figure 11B) causes a distinct loop at high *Ca*^2 + ^ concentrations (trace marked ▾ in Figure 11C). The traces for sarcomere length in Figure 11D reflect the changes in force developed, showing an increased degree of shortening with an enhancement in MCS. Peak shortening velocity also increases with an increase in myofilament *Ca*^2 + ^sensitivity as shown in Figure 11E.

*Effect of Isoproterenol:* The *β*-adrenergic agonist isoproterenol (ISO) is known to cause a decrease in myofilament *Ca*^2 + ^sensitivity as a result of PKA-mediated phosphorylation of troponin I at Ser23/Ser24 [15,17]. However, the increase in amplitude of myoplasmic *Ca*^2 + ^transient more than compensates for the decrease in *Ca*^2 + ^sensitivity in order to facilitate the inotropic effect of *β*-adrenergic stimulation. Here, we adopt a 1 Hz stimulation protocol used by Roof et al. ([40]; Figure 1) to study the effect of 1 *μ*M isoproterenol on isometric contraction and compare it with the effect of increasing extracellular *Ca*^2 + ^ concentration (*Ca*^2 + ^_*o*_) from 1 mM (control) to 3 mM. As shown in Figure 4A, administration of 1 *μ*M isoproterenol or an increase in *Ca*^2 + ^_*o*_to 3 mM causes a substantial (3-fold) enhancement in peak myoplasmic *Ca*^2 + ^_*myo*_ as observed experimentally ([40]; Figure 1). The isoproterenol-dependent effect is a result of PKA mediated dose-dependent increase (23%) in peak I_*Ca,L*_ current together with an increase (17%) in the maximal uptake rate of the SERCA pump when compared to the control case (Appendix, Eqns. 57). In the isoproterenol, the significant increase in peak *Ca*^2 + ^_*myo*_ causes an increase in the strength of isometric tension developed (Figure 4B) despite a decrease in myofilament *Ca*^2 + ^ sensitivity. Increase in *Ca*^2 + ^_*o*_to 3 mM causes a similar effect of increasing isometric tension due to an increase in activator *Ca*^2 + ^ in the cytosol. The increase in peak *Ca*^2 + ^_*myo*_ is a result of an increase in SR *Ca*^2 + ^ content due to enhanced *Ca*^2 + ^ entry via I_*Ca,L*_ assisted by impaired *Na*^ + ^/*Ca*^2 + ^ exchange due to elevated *Ca*^2 + ^_*o*_. However, compared to *β*-adrenergic stimulation, the lack of a decrease in MCS results in a moderately larger developed force. Figure 4C shows the phase plots of self normalized force versus the instantaneous *Ca*^2 + ^concentration in the cytosol. As expected, either the presence of isoproterenol or an increase in *Ca*^2 + ^_*o*_causes rightward extension (due to increase in peak *Ca*^2 + ^_*myo*_) of the phase plot and increases the area enclosed by it due to elevated myoplasmic *Ca*^2 + ^ level combined with an increase in tension developed. In the presence of isoproterenol, the increase in isometric tension developed occurs despite a decrease in myofilament *Ca*^2 + ^ sensitivity (Figure 4C) which manifests as an increase (0.65 (Figure 4C) which manifests as an increase (0.65 *μ*M to 0.85 *μ*M) in EC_50_, the average *Ca*^2 + ^_*myo*_ at 50% of maximal developed force.

## Discussion

Myofilament dynamics have been captured by various representations ranging from the highly simplified models to complex empirical [41,42] and biophysical models [13]. While simplified models tend to use an explicit parabolic tension profile [43], the empirical models use predefined expressions to specify the average force developed by the cross bridges, based on experimental observations of isolated muscle contraction under different loading conditions. On the contrary, biophysical models of cardiac myofilament dynamics include descriptions of cross-bridge cycling and their elastic properties. An extensive review of various myofilament models in the literature is given by Trayanova and Rice [44]. We have developed a composite multiphysics model of excitation-contraction coupling in the rat ventricular myocyte based on a mechanistic electrochemical model of calcium-induced calcium-release (CICR) [4] and a detailed mechanochemical model of cooperative activation and crossbridge cycling [13]. After integrating these component models, the resultant multiphysics model of cardiac electromechanics is used to examine the mechanisms regulating myofilament contractility in an isolated rat ventricular myocyte under a voltage clamp protocol.

In particular, we have studied the role of different modulatory factors in influencing the various types of twitch response (isosarcometric, unloaded and isometric) elicited by an isolated rat ventricular myocyte. The dependence of isosarcometric contraction on the amount of activator *Ca*^2 + ^ available in the myoplasm to trigger contraction and the sarcomere length which modulates myofilament *Ca*^2 + ^ sensitivity is demonstrated (Figure 3). Unloaded cell contraction is investigated to understand the influence of *Ca*^2 + ^_*myo*_ transient on the degree of sarcomere contraction achieved by an unrestrained cell highlighting the enhanced shortening velocity and rate of recovery with increasing peak *Ca*^2 + ^_*myo*_ (shorter time to peak and faster rate of relaxation in the inset in Figure 11B). In agreement with Rice et al. [13] we demonstrate that in isometric contraction (Figure 6), there can be significant internal shortening of the sarcomere as the result of compliant end connections (low KSE values).

*Ca*^2 + ^ released as a result of CICR is known to act as an actuator, triggering myofilament contraction by binding to the low affinity regulatory sites on troponin C which act as the sensor, the *Ca*^2 + ^affinity of which is a function of dynamically changing sarcomere length as well as the fraction of strongly bound crossbridges. This hence facilitates not only a feedforward but also a moderate feedback interaction between the [*Ca*^2 + ^]_*myo*_ transient and the myofilament contractile mechanism. This feedback effect is seen in Figure 8C where the presence of isometric contraction results in a small decrease in peak *Ca*^2 + ^ transient, a result of the *Ca*^2 + ^buffering action of troponin.

Although it is well known that myoplasmic *Ca*^2 + ^concentration has a direct influence on beat to beat cell shortening, other indirect modulatory influences exist. Of particular interest is the indirect coupling of the cellular plasma membrane with the myofilament contractile mechanism. A decrease in *Na*^ + ^/*Ca*^2 + ^ exchanger function in extruding *Ca*^2 + ^from the cytosol results in an increased relative role for the SERCA pump, augmenting SR *Ca*^2 + ^content. This in turn results in enhanced release increasing the availability of post-release activator *Ca*^2 + ^in the cytosol, thus translating into greater degree of contraction (Figure 9). This completes a control loop that allows modulation of NCX activity to force a readjustment in myofilament contractility.

Experimental conditions such as temperature strongly influence cardiac myofilament contractility. An increase in temperature results in an increase in sensitivity of the myofilaments to myoplasmic *Ca*^2 + ^[22]. As the temperature is increased from 22°C to 30°C, increasing myofilament *Ca*^2 + ^sensitivity (Figure 10C) causes a moderate increase in peak force despite a decline in peak *Ca*^2 + ^_*myo*_. However, a further increase to body temperature results in a steep decline in force developed (Figure 10D). Hence, an increase in temperature from 22°C to 38°C which results in a decline in peak *Ca*^2 + ^_*myo*_[14], causes an overall decrease in force developed. This translates into an overall decrease in the degree of cell shortening with increasing temperature (Figure 10F). This temperature dependent behavior is not captured by the model proposed by Rice et al. [13] where an increase in temperature causes an increase in peak force developed as a result of the large Q10 values used (Qf_*app*_, Qh_*f*_, Qh_*b*_ and Qg_*xb*_, Table 1, [13]).

Modulation of myofilament *Ca*^2 + ^sensitivity (MCS) as a result of a change in Troponin I (TnI) phosphorylation by PKA has been implicated in heart failure [45]. Here we study the role of MCS in modulating myofilament contractile response (Figure 11) an aspect overlooked in recent modeling studies including Rice et al. [13]. In particular, we model the effect of isoproterenol, a *β*-adrenergic agonist, which is known to cause a decline in myofilament *Ca*^2 + ^ sensitivity as a result of protein kinase A (PKA) mediated phosphorylation of troponin I at Ser23/Ser24 [15,16]. Such a decline in *Ca*^2 + ^sensitivity aids myofilament relaxation in the presence of increased levels of activator *Ca*^2 + ^. Figure 4 shows that an increase in amplitude of the myoplasmic *Ca*^2 + ^transient (a cumulative effect of enhancement in trigger current and increase in uptake rate of the SERCA pump) is more than adequate to compensate for the decrease in *Ca*^2 + ^ sensitivity, thus facilitating the desired effect of *β*-adrenergic stimulation, namely an increase in contractile force generated.

### Model limitations

1. Our model of a rat ventricular myocyte is limited to *Ca*^2 + ^related channels, exchanger and pumps (*I*_*Ca,L*_, *I*_*NaCa*_, *I*_*PMCA*_, *I*_*ryr*_and SERCA pump), while lacking exclusive *Na*^ + ^or *K*^ + ^related channels and transporters (described in Krishna et al. [4]). In our voltage clamp protocol, external solution in the bath is modeled as normal Tyrode with *Cs*^ + ^substituted for *K*^ + ^. We have represented *I*_*NaCs*_in our model (described in Krishna et al. [4]) by the expression for *Na*^ + ^/*K*^ + ^pump formulated by Lindblad et al. [46] replacing *K*^ + ^ion concentrations with the *Cs*^ + ^ion concentration. While ensuring whole cell *Na*^ + ^ion balance, the peak *Na*^ + ^/*Cs*^ + ^pump current is modified to be one-sixth to account for the decreased potency of the cation *Cs*^ + ^in activating the pump. The voltage-dependence of *I*_*NaCs*_is adopted from the data on *Na*^ + ^/*K*^ + ^pump from Hansen et al. [47]. The role of mitochondrial *Ca*^2 + ^uniporter is not modeled in this study due to its negligible (< 1%) role in *Ca*^2 + ^transport from the cytosol [48]. This model is aimed at mimicking voltage clamp conditions where channels other than calcium are blocked, and it cannot be used to study any action potential-induced *Ca*^2 + ^transient-frequency relationship. However, its focus on the *Ca*^2 + ^dynamics allows one to comprehend more clearly the important role of *Ca*^2 + ^signalling pathways and feedback control systems in maintaining whole cell homeostasis over a prolonged period of time.

2. The cooperative activation of the thin filament and the strain-dependent transitions of the crossbridge cycle have been approximately modeled as non-spatial, state-variables. However, this simplification is valid as these transitions are inherently local phenomena and the model reproduces a wide range of steady state and dynamic responses in cardiac muscle [13]. Although a CaM-dependent pathway is reported [18] to be responsible in modulation of myofibrillar contractility implying a possible CaM mediated role for Ca-dependent kinases or phosphatases in regulating myofilament contractility (particularly in frequency dependent acceleration of relaxation), further studies are required to clarify the molecular mechanisms involved.

3. The temperature dependence of passive force attributed to titin and other cytoskeletal elements is not modeled in this study and the assumption of constant stiffness for the series elastic element (KSE) does not account for temperature dependent effects. This is an area where the model can be expanded but additional measured data on the temperature sensitivity of these elements is necessary. Regardless, our model provides reasonable approximations to the temperature dependence of developed force in intact thin rat ventricular trabeculae [14].

## Conclusion

We have developed a composite mathematical model for cardiac electromechanics which includes a detailed description of *Ca*^2 + ^dynamics under voltage clamp conditions in the rat ventricular myocyte, based on experimental data [4,13]. We have investigated the role of different factors including the myoplasmic *Ca*^2 + ^_*myo*_ transient and the sarcomeric length in influencing various types of twitch responses obtained under different loading conditions (including isosarcometric, isometric and unloaded conditions). Various control loops influencing cell shortening have been explored. In particular, the bidirectional interaction of the *Ca*^2 + ^ transient with the myofilament contractile mechanism and the importance of indirect SR mediated interaction of the sarcolemma with the contractile machinery is highlighted by showing the regulation of isometric contraction by the degree of NCX activity. The effect of temperature on cell shortening is investigated identifying the differential sensitivity of the *Ca*^2 + ^_*myo*_ transient and the myofilament contractile mechanism. The important role of myofilament *Ca*^2 + ^sensitivity in force generation is studied with particular emphasis on the effect of *β*-adrenergic stimulation on cardiac contractile response. In agreement with Janssen [37], we also demonstrate a key linear relationship between the rate of contraction and relaxation, which is shown here to be intrinsically coupled over the full range of physiological perturbations (including temperature, sarcomeric length, activator *Ca*^2 + ^, and isoproterenol; e.g. see insets in Figures 10D, 3A-ii).

This study demonstrates that the model has long-term stability in regulating myoplasmic *Ca*^2 + ^, as shown in the 18-sec duration experiments at a physiological rate of stimulation (conditioning train of voltage clamp pulses at 5 Hz) shown in Figure 9. This long term *Ca*^2 + ^balance under physiological conditions is crucial in facilitating implementation of this model in large scale simulations such as frequency dependent studies analyzing cellular force-frequency response. Our study also provides mechanistic insights into whole-cell responses to a wide variety of testing approaches used in studies of cardiac myofilament contractility that have appeared in the literature over the past two decades (Figures 3,4,5,6,7,8,9,10 and 11). Thus, the model serves as a platform for the predictive modeling of VC investigations of cardiac electromechanics pertaining to the rat ventricular myocyte in a number of areas. These are fundamental issues that would benefit from a better mechanistic understanding of the cardiac contractile mechanism in the rat ventricluar myocyte. This study is aimed at providing an initial step towards this goal.

## Appendix

### Equations governing electro-mechanics modified (from Rice et al. [13]) in the model

Regulatory Ca^2 + ^-binding to troponin

(1) $$\begin{array}{ll} \frac{\mathrm{dCaTro}p_{H}}{\mathrm{dt}}= & k_{\text{onT}}\mathrm{Tn}I_{u}{\left[Ca^{2+}\right]}_{\text{myo}}(1-\mathrm{CaTro}p_{H})\\ & -k_{\text{offHT}}\mathrm{CaTro}p_{H} \end{array}$$

(2) $$\begin{array}{ll} \frac{\mathrm{dCaTro}p_{L}}{\mathrm{dt}}= & k_{\text{onT}}\mathrm{Tn}I_{u}{\left[Ca^{2+}\right]}_{\text{myo}}(1-\mathrm{CaTro}p_{L})\\ & -k_{\text{offLT}}\mathrm{CaTro}p_{L} \end{array}$$

k_*onT*_ = 22.22 *μ*M^−1^s^−1^; k_*offHT*_ = 17.36 s^−1^; k_*offLT*_ = 173.61 s^−1^; (Rice et al. [13]).

(3) $$\begin{array}{ll} \frac{\mathrm{dTn}I_{p}}{\mathrm{dt}}= & k_{\text{onTI}}\Delta_{\text{PKA}}\mathrm{Tn}I_{u}{-}_{\text{offTI}}\mathrm{Tn}I_{p} \end{array}$$

k_*onTI*_ = 698.69 s^−1^ ; k_*offTI*_ = 80.0 s^−1^; (estimated from Roof et al. [40]).

(4) $$\begin{array}{c} \mathrm{Tn}I_{u}=1-\mathrm{Tn}I_{p} \end{array}$$

(5) $$\begin{array}{c} \Delta_{\text{PKA}}=\frac{0.3\times [\mathrm{ISO}]}{[\mathrm{ISO}]+12.1} \end{array}$$

*ISO* - concentration of isoproterenol in *μ*M.

(6) $$\begin{array}{c} \mathrm{PK}A_{\text{act}}=0.1+\Delta_{\text{PKA}} \end{array}$$

*PKA*_*act*_ - Relative regulatory activity of PKA (unitless).

(7) $$\begin{array}{c} P_{\text{Ca}}=10.0\times \mathrm{PK}A_{\text{act}}P_{\text{Ca}}^{\text{old}} \end{array}$$

$P_{\text{Ca}}^{\text{old}}$ = 4.5408 *μ*L s^−1^(Krishna et al. [4]).

## Abbreviations

[Ca2+]: calcium ion concentration; [Ca2+]myo: myoplasmic Ca2+ concentration; [Ca2+]o: extracellular Ca2+ concentration; CaM: calmodulin; CICR: calcium-induced calcium-release; CRCP: contraction-relaxation coupling point; DCU: dyadic coupling unit; DHP: dihydropyridine; E-C: excitation contraction; EC50: half maximal effective concentration; F-Ca: force versus Ca2+; FFR: force frequency response; FRCRSFa: Phe-Arg-Cys-Arg-Ser-Phe-CONH2; F-SL: force versus sarcomere length; ICa,L: L-type Ca2+ current; INaCa: sodium calcium exchanger current; IPMCA: plasma membrane Ca2+ ATPase pump current; ISO: Isoproterenol; jSR: junctional portion of the sarcoplasmic reticulum; KSE: stiffness coefficient of the non-contractile series elastic element; LSR: longitudinal portion of the sarcoplasmic reticulum; L-type: long lasting type; MCS: myofilament Ca2+ sensitivity; μm: micro meter; μM: micro molar; mM: milli molar; mV: milli volt; NCX: Na+/Ca2+ exchanger; ODE: ordinary differential equation; pA/pF: pico amps per pico farad; PKA: protein kinase A; RT50: time required for 50% sarcomere relaxation; RT50,C: time required for 50% decline in Ca2+-transient; RT50,F: time required decline in force response; RT50,I: time required for 50% ICa,L inactivation; RyR: receptor; SERCA: sarcoplasmic reticulum Ca2+ ATPase; SL: sarcomere length; SR: sarcoplasmic reticulum; TD50: time taken from 50% activation to 50% relaxation; TnC: Troponin C; TnI: Troponin I; TnIu: fraction of unphosphorylated Troponin I; TTP: time required to attain peak value;.

## Competing interests

The authors declare that they have no competing interests.

## Authors’ contributions

AK developed the coupled electromechanical model of the rat ventricular myocyte, carried out the voltage clamp modeling studies, and drafted the manuscript. MV made substantial intellectual contributions to the study and in drafting of the manuscript. PTP made intellectual contributions to the manuscript as well as significant contributions to the drafting of the manuscript. JWC made key contributions to the conception of the study, design, analysis and interpretation of results, and drafting of the manuscript. All authors read and approved the final manuscript.
